# Efficacy of transcranial magnetic stimulation for mild cognitive impairment: a systematic review and meta-analysis of randomized controlled trials

**DOI:** 10.3389/fneur.2026.1788223

**Published:** 2026-05-18

**Authors:** Yongqiang Lin, Yongjun Tao, Zixin He, Huaqiang Wang, Yongfei Zheng, Yue Jin

**Affiliations:** 1 The First People's Hospital of Wenling, Affiliated Wenling Hospital, Wenzhou Medical University, Wenling, Zhejiang, China; 2 Lishui Hospital of Traditional Chinese Medicine Affiliated to Zhejiang University of Chinese Medicine, Lishui, Zhejiang, China

**Keywords:** cognition impairment, cognition, MCI (mild cognitive impairment), meta-analysis, TMS (repetitive transcranial magnetic stimulation)

## Abstract

**Background:**

Transcranial magnetic stimulation (TMS) has been explored as a non-invasive intervention for cognitive impairment in mild cognitive impairment (MCI), but evidence regarding its overall efficacy remains inconsistent. This meta-analysis evaluated the pooled effect of TMS on cognitive outcomes in patients with MCI.

**Methods:**

Randomized controlled trials (RCTs) enrolling participants with MCI only were identified from PubMed, CENTRAL, and Embase through November 10, 2025, in accordance with PRISMA and a PROSPERO-registered protocol (CRD420251274531). Cognitive outcomes were pre-specified and synthesized by domain, including global cognition and executive function (primary focus), as well as memory, language/naming, and neuropsychiatric measures. Pooled effects were calculated using fixed- or random-effects models as appropriate and reported as mean differences (*MD*) or standardized mean differences (SMDs) calculated as Hedges'g with 95% confidence intervals. Risk of bias, methodological quality, and certainty of evidence were assessed using RoB 1, PEDro, and GRADE.

**Results:**

Eleven RCTs (532 participants with MCI) were included. rTMS improved global cognition on MMSE (*MD* = 2.35, 95% CI 1.74–2.96; *k* = 5, *n* = 120) and MoCA (*MD* = 1.31, 95% CI 0.80–1.82; *k* = 6, *n* = 337). Verbal memory also improved in AVLT-based outcomes (delayed recall: *k* = 5, *n* = 125; recognition: *k* = 4, *n* = 103). Attention/executive measures favored rTMS, including Digit Span (*MD* = 0.62, 95% CI 0.37–0.87) and shorter completion times on Trail Making Test-A (*MD* = −3.33, 95% CI −3.76 to −2.90) and TMT-B (*MD* = −4.08, 95% CI −7.92 to −2.23). Naming ability and neuropsychiatric outcomes also improved, whereas language fluency showed no significant between-group difference. Overall risk of bias was low; however, due to limited sample sizes, GRADE certainty was mostly moderate.

**Conclusion:**

In MCI-only RCTs, rTMS improved global cognition (MMSE/MoCA) and AVLT-based verbal memory (delayed recall/recognition). Selected executive/attention, naming, and neuropsychiatric outcomes also improved, whereas language fluency was unchanged. Evidence certainty was mostly moderate, and larger high-quality RCTs are needed.

**Systematic review registration:**

https://www.crd.york.ac.uk/PROSPERO/view/CRD420251274531, identifier: CRD420251274531.

## 1 Introduction

Dementia has emerged as a major global public health challenge, imposing substantial psychological, social, and economic burdens on affected individuals, their caregivers, and society as a whole (1). Mild cognitive impairment (MCI) refers to a level of mental decline that exceeds what would be expected for an individual's age and educational background and may involve multiple cognitive domains, including memory, language, attention, visuospatial abilities, and executive function (2). At the same time, daily functional independence is largely preserved. In clinical and research settings, MCI is commonly operationalized using established diagnostic frameworks, including the Petersen criteria, the National Institute on Aging–Alzheimer's Association (NIA-AA) criteria, and the DSM-5 construct of mild neurocognitive disorder (3–5). MCI is widely regarded as an intermediate clinical state between normal aging and dementia, characterized by objective cognitive impairment that exceeds age- and education-adjusted expectations while functional independence is largely preserved. Importantly, MCI is a heterogeneous syndrome with multiple possible trajectories. A proportion of individuals, particularly those with amnestic presentations, may represent a prodromal stage of Alzheimer's disease (AD) or other neurodegenerative conditions (6). In contrast, others remain stable for years or revert to normal cognition (7, 8). Epidemiological evidence indicates that the prevalence of MCI among adults aged 60 years and older is approximately 12 to 18%, and that affected individuals have an elevated risk of progression to dementia, with reported annual progression rates of roughly 10 to 18% (9, 10). Although independence in basic activities of daily living is typically preserved in MCI by definition, individuals may still experience meaningful reductions in quality of life due to cognitive complaints, emotional distress, or subtle functional difficulties in complex daily tasks (11, 12).

Although AD has several pharmacological treatments, therapeutic response is inconsistent, and implementation in routine care can be limited by eligibility requirements, cost, and availability, as well as the need for safety surveillance (13). By contrast, MCI still lacks a clearly established disease-modifying medication, and drug-based management at this stage remains uncertain (14–16). As a result, care for MCI is largely built around non-pharmacological strategies, such as strengthening cognitive reserve, increasing physical activity, and providing cognitive training (17, 18). However, reported benefits are typically modest and variable, and maintaining long-term participation and access to structured programs can be difficult in real-world settings. Together, these challenges motivate the exploration of adjunctive interventions that are less reliant on sustained behavioral engagement and that can directly influence the neural networks disrupted in early cognitive decline. In line with this need, repetitive transcranial magnetic stimulation (rTMS), including intermittent theta burst stimulation (iTBS), is a non-invasive neuromodulation approach that can engage large-scale cognitive control networks and has garnered increasing attention as a potential intervention for MCI.

TMS is a non-invasive neuromodulation technique capable of modulating cortical excitability and large-scale brain networks through electromagnetic induction (19–21). rTMS, in particular, can induce lasting neuroplastic effects beyond the stimulation period, which are considered critical for its therapeutic potential (22, 23). Based on these properties, rTMS has been increasingly explored as an intervention for cognitive impairment, including MCI and early AD. Several randomized controlled trials (RCTs) and subsequent systematic reviews have evaluated its effects on cognitive outcomes, reporting potential benefits but also substantial heterogeneity in stimulation targets, protocols, and outcome measures. Importantly, many prior reviews pooled MCI with early AD or relied primarily on global cognitive scales, which limits inference on domain-specific responsiveness within MCI (24, 25). This leaves it unclear whether rTMS preferentially benefits specific cognitive domains (e.g., executive control) in MCI. As a result, the magnitude, domain specificity, and consistency of rTMS-related cognitive improvements in individuals with MCI remain uncertain.

Although rTMS has demonstrated good safety and feasibility in cognitive interventions, its therapeutic efficacy in MCI remains controversial. Several RCTs have reported that high-frequency rTMS, most commonly delivered over pre-frontal regions such as the dorsolateral pre-frontal cortex (DLPFC), may improve global cognition and executive function in individuals with MCI, whereas effects on memory and language outcomes have been inconsistent or non-significant (26, 27). Existing systematic reviews and meta-analyses have attempted to synthesize these findings and generally suggest potential benefits; however, their conclusions are constrained by heterogeneity in diagnostic composition (e.g., pooling MCI with early AD), stimulation protocols/targets, and outcome measures, as well as small sample sizes and variable methodological quality (24, 25). Importantly, because MCI represents a prodromal stage with distinct baseline performance and treatment responsiveness, mixing it with dementia spectra can obscure effects and weaken clinical interpretability for MCI.

To move beyond prior syntheses, we conducted a systematic review and meta-analysis restricted to RCTs enrolling participants with MCI only and performed a domain-specific synthesis to delineate which cognitive functions respond to rTMS rather than relying primarily on global cognitive scales. In contrast to earlier reviews that emphasized overall cognition, we pre-specified a mechanistically informed hypothesis: given that most MCI rTMS trials target pre-frontal control networks implicated in executive control, we expected rTMS to yield larger and more consistent improvements in executive function than in global cognition or other domains. This framework guided outcome selection and interpretation in the present study.

## 2 Methods

This review adhered to the Preferred Reporting Items for Systematic Reviews and Meta-Analyses (PRISMA) guidelines and was prospectively registered with PROSPERO (CRD420251274531).

### 2.1 Search strategy

The review followed a three-stage process of identification, screening, and inclusion. RCTs published from database inception to November 10, 2025, were systematically searched in PubMed, Embase, and the Cochrane Library (CENTRAL), with eligibility limited to English-language studies. The search strategy comprised disease-related terms (e.g., MCI), intervention-related terms (e.g., non-invasive brain stimulation, transcranial magnetic stimulation, theta burst stimulation, and non-invasive neuromodulation), and study design terms for RCTs. Controlled vocabulary (such as MeSH terms) and free-text keywords were used, with truncation symbols applied to capture term variations. Reference lists of eligible studies were manually screened, and authors of conference abstracts were contacted when necessary. The complete search strategy is provided in Supplementary Table 1.

### 2.2 Inclusion and exclusion criteria

Two investigators (Y.L. and Y.J.) independently identified and screened RCTs evaluating rTMS as an active intervention. Eligible participants were individuals diagnosed with MCI (any subtype; e.g., amnestic or non-amnestic, single- or multi-domain) according to established diagnostic criteria; studies involving patients with early AD were excluded.

#### 2.2.1 Inclusion criteria were as follows

(1) Participants diagnosed with MCI (any subtype; e.g., amnestic or non-amnestic, single- or multi-domain) according to established diagnostic criteria (e.g., Petersen criteria or NIA-AA criteria; DSM-5 mild neurocognitive disorder when used to operationalize MCI), without meeting criteria for AD;

(2) RCTs employing an eligible randomized design;

(3) rTMS delivered as the active intervention (including conventional rTMS and iTBS);

(4) At least one standardized neuropsychological cognitive outcome reported;

(5) Studies conducted in human participants and published in English.

#### 2.2.2 Exclusion criteria included

(1) Non-original publications (e.g., reviews, meta-analyses, editorials, conference abstracts, case reports, or protocols);

(2) Studies involving patients with Alzheimer's disease (including early AD) or other neurodegenerative disorders;

(3) Cognitive impairment attributable to other neurological conditions (e.g., Parkinson's disease or stroke);

(4) TMS was not applied as the primary intervention;

(5) Insufficient data for the extraction of cognitive outcomes.

### 2.3 Study selection and data extraction

Two reviewers (Y.L. and H.W.) independently screened all retrieved records using pre-defined eligibility criteria. References were managed with EndNote (Clarivate Analytics) to remove duplicates. Titles and abstracts were screened initially, followed by full-text review of potentially eligible studies for final inclusion. Disagreements were resolved by discussion or, when necessary, adjudicated by a third reviewer (Y.Z.).

Data extraction was independently conducted by two investigators (Y.L. and H.W.) using a pre-defined standardized extraction form, with discrepancies resolved through discussion. Information collected included the first author, publication year, country, study design, sample size, details of the intervention and control conditions, primary outcome measures, and follow-up duration. For cognitive outcomes, mean values and standard deviations (SDs) were directly extracted from tables in most studies; in two studies where results were presented graphically, data were digitized using GetData Graph Digitizer (http://getdata-graph-digitizer.com). When relevant data were not explicitly reported, corresponding authors were contacted, and if unavailable, values were estimated according to the recommendations of the Cochrane Handbook for Systematic Reviews of Interventions (version 5.1.0, Section 16.1.3.2).

All data were independently checked by two reviewers (Y.L. and H.W.), with disagreements resolved by consensus or adjudication by a third reviewer (Y.Z.). Inter-rater agreement, assessed using Cohen's kappa, was high across screening and extraction stages (κ = 0.83 for title/abstract screening, 0.86 for full-text review, and 0.79 for data extraction).

#### 2.3.1 Data extraction items

In addition to outcome data, we pre-specified and extracted the following non-outcome variables to characterize included trials and rTMS protocols and to support interpretation of between-study heterogeneity: (1) participant characteristics (mean age, sex distribution, diagnostic criteria for MCI, and baseline cognitive status where available); (2) intervention characteristics (coil type, stimulation target/site, stimulation frequency, stimulation intensity (e.g., % resting motor threshold), number of pulses per session, number of sessions and total treatment duration, and the use of neuro-navigation if reported); and (3) comparator characteristics (type of sham or control condition and co-interventions). These items correspond to the study characteristics summarized in Table 1.

**Table 1:** Baseline characteristics of the included studies.

Study	Country	Design	rTMS group										Control group				Adverse effect	Outcome	Measurement timepoint (Week)
			Sample size	Age (year)	Female (%)	Device/ Coil type	Stimulation site	Target	Stimulation Frequency type	Stimulation frequency/ intensity	Sessions per week	Total sessions/ pulses	Sample size	Age (year)	Female (%)	Intervention			
Cirillo et al. (30)	Italy	RCT, 2 arms	10	66.5 ± 3.94	60.00%	NR	Bilateral rTMS	DLPFC	High-frequency rTMS	10 Hz, NR	1time/day	28 sessions/NR	10	70.5 ± 5.15	60.00%	Sham rTMS	Mild stimulation-site discomfort (*n* = 3) and mild headache (*n* = 2) were reported; no significant adverse events occurred	RBANS, BDI-II, BAI, AES	4 weeks, 24 weeks
Cui et al. (31)	China	RCT, 2 arms	11	73.91 ± 10.01	72.73%	MagPro R30 (MagVenture) Figure-of-eight coil (MCF-B70, 97 mm)	Right DLPFC	DLPFC	High-frequency rTMS	10 Hz, 90% RMT	1time/day	10 sessions/15,000 pulses	10	74.00 ± 7.62	50.00%	Sham rTMS	Not reported	MMSE, ACE-III, AVLT	2 weeks, 8 weeks
Drumond Marra et al. (32)	Brazil	RCT, 2 arms	15	65.1 ± 3.5	60.00%	MagPro X100(MagVenture A/S, Farum, Denmark)Figure-of-eight coil	Left DLPFC	DLPFC	High-frequency rTMS	10 Hz, 110% RMT	1time/day	10 Sessions/20,000 pulses	19	65.2 ± 4.1	68.40%	Sham rTMS	Reported None	MoCA, HAMD, HAMA	2 weeks, 4 weeks
Esposito et al. (33)	Italy	RCT, 3 arms	11	64.00 ± 13.00	54.80%	Magstim2 Rapid (The Magstim Company, UK) Standard figure-of-eight coil	Bilateral rTMS	DLPFC	High-frequency rTMS	10 Hz, 80% RMT	1time/day	20 Sessions/40,000 pulses	16	70.05 ± 11.00	55.50%	Sham rTMS	Not Reported	BDI-II, BAI, AES	1 week
Fu (34)	China	RCT, 2 arms	98	64.17 ± 4.33	50.00%	MagPro-R30 magnetic stimulator (Tonica, Denmark) Not explicitly specified	Bilateral rTMS	Bilateral prefrontal cortex	High-frequency rTMS	10 Hz, 80% RMT	1time/day	40 Sessions/100,000 pulses	82	63.57 ± 3.59	43.90%	Sham rTMS	Diarrhea (*n* = 4), nausea and vomiting (*n* = 10), and mild dizziness (*n* = 12) were reported	WMS–RC, MoCA	8 weeks
Liu et al. (35)	China	RCT, 2 arms	55	67.8 ± 4.5	67.30%	Rapid2 stimulator (Magstim)Standard 70 mm figure-of-eight coil	Right DLPFC	DLPFC	Low-frequency rTMS	1 Hz, 80% RMT	1time/day	30 Sessions/36,000 pulses	55	68.0 ± 4.6	56.40%	Sham rTMS	Not Reported	PSQI, MoCA, HAMA, HAMD	6 weeks, 12 weeks
Padala et al. (36)	USA	RCT, 2 arms	5	64.0 ± 9.0	20.00%	NeuroStar^®^ TMS Therapy SystemXPLOR standard treatment coil	Left DLPFC	DLPFC	High-frequency rTMS	10 Hz, 120% RMT	1time/day	10 Sessions/30,000 pulses	4	68.0 ± 10.0	0.00%	Sham rTMS	Treatment-site discomfort (*n* = 8), shock sensation (*n* = 3), facial twitching (*n* = 1), insomnia (*n* = 1), and dizziness (*n* = 1)	MMSE, TMT	2 weeks, 4 weeks
Song et al. (36)	China	RCT, 2 arms	11	65.55 ± 3.98	45.45%	MagPro R30 magnetic stimulator (Denmark Tonica Company) MCFB65	Left DLPFC	DLPFC	High-frequency rTMS	10 Hz, 90% RMT	1time/day	10 Sessions/15,000 pulses	11	69.27 ± 4.38	63.64%	Sham rTMS	Not Reported	MMSE, MoCA, AVLT, BNT, AFT	2 weeks
Wang et al. (37)	China	RCT, 2 arms	14	69.63 ± 6.38	64.29%	CCY-II stimulator (Yiruide Medical Equipment Co., Wuhan, China) Figure-of-eight	Right DLPFC	DLPFC	iTBS	50 Hz, 80% RMT	1time/day	10 Sessions 6,000 pulses	13	69.86 ± 6.80	69.23%	Sham iTBS	Mild headache, dizziness, and facial twitching (each *n* = 1); no serious adverse events	MoCA, TMT, DST	2 weeks
Wang et al. (38)	China	RCT, 2 arms	19	65.63 ± 4.28	57.89%	MagPro R30 (MagVenture, Denmark) 70-mm butterfly-shaped coil	Left DLPFC	DLPFC	High-frequency rTMS	10 Hz, 90% RMT	1time/day	10 Sessions/15,000 pulses	20	68.45 ± 5.19	75.00%	Sham rTMS	Transient dizziness (*n* = 2), mild discomfort	MoCA, AVLT, MMSE	2 weeks
Zheng et al. (39)	China	RCT, 4 arms	14	67.00 ± 10.95	50.00%	YIRUIDE CCY-IV stimulator (Yiruide Co., Ltd., Wuhan, China) Standard figure-of-eight	non-DLPFC, parietal target	non-DLPFC, parietal target	iTBS	50 Hz, 70% RMT	1time/day	20 Sessions/12,000 pulses	6	65.00 ± 5.75	53.85%	Sham iTBS	Mild stimulation-site headache (*n* = 5); no serious adverse events	MoCA, AVLTMMSE, TMT	4 weeks
Zheng et al. (39)	China	RCT, 4 arms	16	66.56 ± 6.68	68.75%	YIRUIDE CCY-IV stimulator (Yiruide Co., Ltd., Wuhan, China) Standard figure-of-eight	Non-DLPFC, parietal target	Non-DLPFC, parietal target	High-frequency rTMS	20 Hz, 100% RMT	1time/day	20 Sessions/32,000 pulses	7	65.00 ± 5.75	53.85%	Sham rTMS	Mild stimulation-siteheadache (*n* = 5); no serious adverse events	MoCA, AVLT, MMSE, TMT	4 weeks

rTMS, repetitive transcranial magnetic stimulation; RCT, randomized controlled trial; NR, not reported; DLPFC, dorsolateral pre-frontal cortex; RMT, resting motor threshold; RBANS, repeatable battery for the assessment of neuropsychological status; BDI-II, Beck Depression Inventory-II; BAI, Beck Anxiety Inventory; AES, Apathy Evaluation Scale; MMSE, mini-mental state examination; MOCA, montreal cognitive assessment; ACE-III, addenbrooke's cognitive examination-III; AVLT, auditory verbal learning test; HAMD, Hamilton Depression Rating Scale; HAMA, Hamilton Anxiety Rating Scale; WMS–RC, Wechsler Memory scale–revised, Chinese version; PSQI, Pittsburgh Sleep Quality Index; TMT, trail making test; DST, digit span test; BNT, boston naming test; AFT, animal verbal fluency test; iTBS, intermittent theta burst stimulation.

Total pulses were extracted as reported in each trial or calculated as pulses per session × total sessions when sufficient protocol details were available; items not reported were labeled as NR.

#### 2.3.2 Management of missing data and reporting inconsistencies

If trial reports were incomplete or unclear, we applied pre-defined, data-type–specific rules. For outcome data, we contacted corresponding authors when key statistics were missing; if unavailable, SDs were derived from SEs, confidence intervals, *t*-values, or *p*-values following Cochrane guidance, and figure-only data were digitally extracted when necessary. For study characteristics and rTMS protocol details (e.g., coil type, target/site, frequency, intensity, pulses, and dose), we sought author clarification; otherwise, items were coded as Not reported (NR) and shown in Table 1. Unreported stimulation parameters were not imputed or inferred.

### 2.4 Assessment of risk of bias

Study quality was assessed using the Cochrane Risk of Bias tool, which evaluates bias related to random sequence generation, allocation concealment, blinding of participants and personnel, blinding of outcome assessment, incomplete outcome data, selective reporting, and other potential sources of bias. Because non-pharmacological interventions such as rTMS are particularly susceptible to performance and detection bias, special attention was paid to blinding procedures and the characteristics of control conditions (e.g., sham stimulation or usual care).

For the selective reporting domain, trial registration records, published protocols, and statistical analysis plans were examined when available. If no pre-registration or protocol information was available and selective reporting could not be ruled out, this domain was judged as having unclear risk rather than low risk. Assessments were conducted independently by two reviewers (Y.L. and Z.H.), with disagreements resolved by a third reviewer (Y.Z.). Methodological quality was additionally evaluated using the Physiotherapy Evidence Database (PEDro) scale as a complementary measure.

### 2.5 Outcome measures

Outcomes were pre-specified and grouped into global cognition and domain-specific cognitive domains (Supplementary Figure 1). Global cognition was assessed using the Mini-Mental State Examination (MMSE) and the Montreal Cognitive Assessment (MoCA), with higher scores indicating better performance.

Domain-specific outcomes included executive function/attention (Digit Span; Trail Making Test [TMT]-A and -B), memory (Auditory Verbal Learning Test [AVLT], including immediate recall, delayed recall, and recognition), and language-related abilities (verbal fluency and naming tasks) (28). Neuropsychiatric outcomes (e.g., depression/anxiety measures) were extracted when reported. For time-based tests (e.g., TMT), shorter completion times indicate better performance, and effect directions were standardized so that values consistently reflected improvement (29). When the same instrument was used across trials, we pooled effects as mean differences; otherwise, standardized mean differences were used. Contributing trials for each outcome are summarized in Supplementary Table 3.

### 2.6 Certainty of evidence assessment

Certainty of evidence for each outcome was assessed using the GRADE methodology with the GRADEpro GDT platform (McMaster University). Under this framework, evidence from RCTs was initially rated as high certainty and was then downgraded by one or two levels, as appropriate, for concerns related to risk of bias, inconsistency, indirectness, imprecision, or reporting bias. Certainty was evaluated separately for each outcome based on the body of evidence contributing to that specific pooled analysis. In the present review, downgrading was mainly driven by imprecision when the total sample size was small and/or confidence intervals were wide, and by inconsistency when substantial between-study heterogeneity was observed. Because fewer than 10 studies contributed to each outcome, publication bias was not formally assessed and was therefore not used as a basis for downgrading unless there was clear suspicion of reporting bias. Consolidated outcome-specific certainty ratings and reasons for downgrading were summarized in the Summary of Findings table generated using GRADEpro.

### 2.7 Meta-analysis

Meta-analyses were conducted in RevMan 5.4. Effect sizes were calculated from post-intervention means and standard deviations together with sample sizes in the intervention and control groups. Continuous outcomes were pooled as mean differences (MDs) or standardized mean differences calculated as Hedges'g with 95% confidence intervals (CIs), using MDs when identical scales were applied and Hedges'g when different instruments were used to assess the same construct, given its small-sample correction. For outcomes where lower scores indicate better performance (e.g., TMT completion time), effect directions were standardized so that values consistently reflected improvement. When a trial included more than two arms, relevant comparisons were included without double-counting participants (e.g., a three-arm trial contributing two comparisons with the shared group appropriately handled). Statistical heterogeneity was assessed using Cochran's Q test and the *I*^2^ statistic.

For outcomes measured using the same scale, a fixed-effect model was used when heterogeneity was low (*I*^2^ ≤ 50%), whereas a random-effects model was used when heterogeneity was substantial (*I*^2^ > 50%). For outcomes synthesized across different assessment instruments, a random-effects model was used to account for potential between-study variability. Results were presented as forest plots, and a two-sided *P*-value ≤ 0.05 was considered statistically significant.

Publication bias. Although 11 RCTs were included overall, each outcome-specific meta-analysis included fewer than 10 studies (*k* < 10); therefore, publication bias was not assessed (e.g., funnel plots or Egger/Begg tests), consistent with Cochrane Handbook guidance.

### 2.8 Sensitivity analysis

Sensitivity analysis was performed for the primary outcome (global cognitive performance). Robustness was evaluated using a leave-one-out approach, recalculating the pooled effect after omitting each study in turn. In addition, because SDs were digitized from figures in two trials, we conducted a supplementary analysis excluding these trials to assess the impact of digitized data on the pooled estimate.

## 3 Results

### 3.1 Study selection process

**Figure 1:**
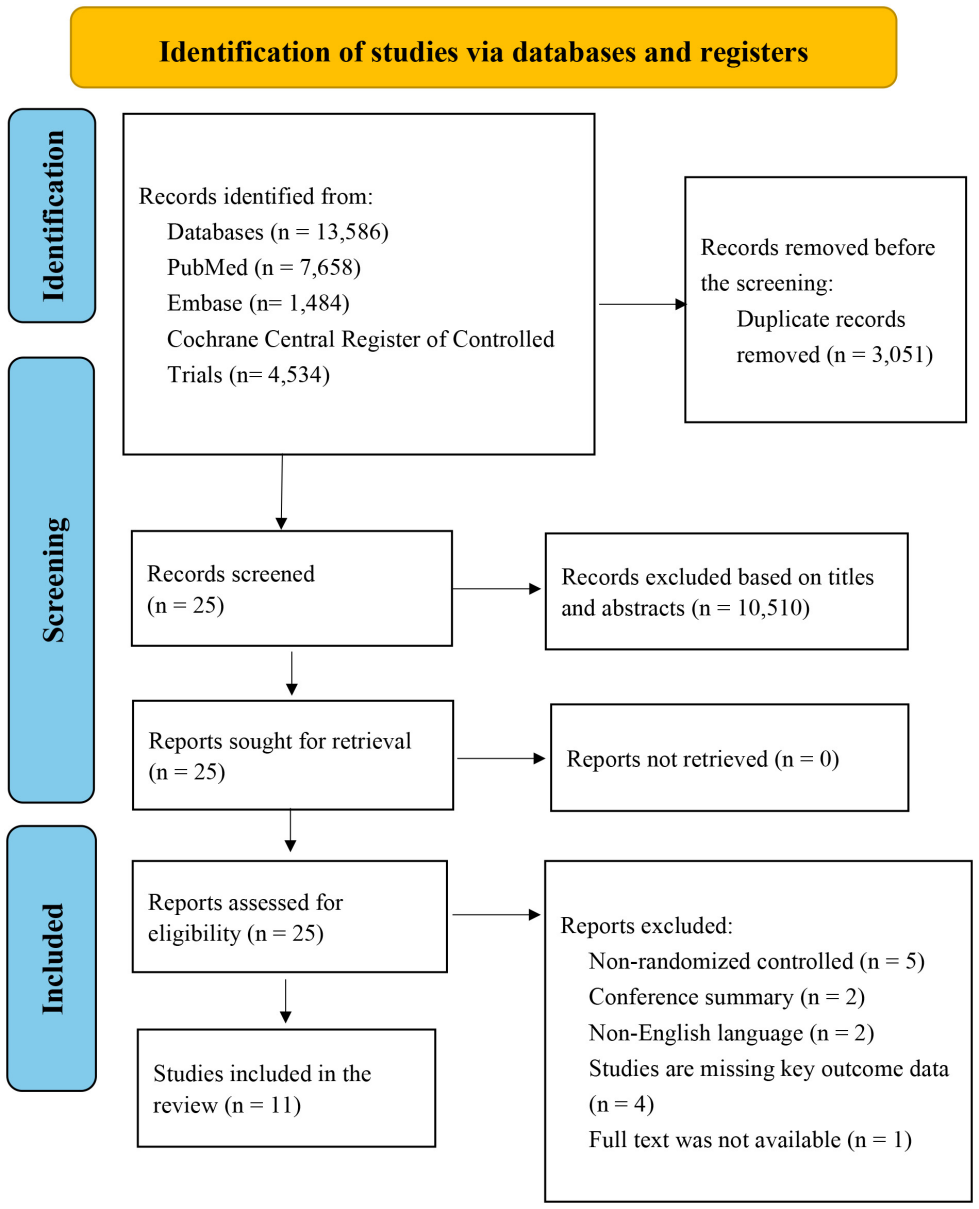
Flowchart of the study selection process.

The study selection process is presented in Figure 1. In total, 13,586 records were identified from databases and trial registries. After removing duplicates, 10,535 records underwent title and abstract screening, and 25 studies were assessed in full text. Of these, 14 were excluded after full-text review due to non-randomized design (*n* = 5), conference abstract only (*n* = 2), non-English-language publication (*n* = 2), missing primary outcomes/insufficient outcome data (*n* = 4), or unavailable full text (*n* = 1); full references (including titles) for all full-text–excluded reports and the primary reasons for exclusion are provided in Supplementary Table 2. Consequently, 11 RCTs were included in the final analysis, with no crossover designs identified.

### 3.2 Study characteristics

A total of 11 studies were included in this meta-analysis. One three-arm trial was split into two independent comparisons, resulting in 12 randomized controlled comparisons (26, 30–39). Regarding diagnostic criteria, two studies enrolled patients with amnestic mild cognitive impairment (aMCI) only (31, 39), whereas the remaining studies recruited MCI without restricting subtype; thus, aMCI cases may have been included, but subtype-specific data were not reported or were not separable. In terms of stimulation modality, most trials delivered conventional rTMS protocols (pre-dominantly high-frequency, with one low-frequency protocol), and two trials used iTBS as the stimulation pattern (37, 39). Stimulation targets pre-dominantly involved the dorsolateral pre-frontal cortex (DLPFC), with four studies targeting the left DLPFC (32, 35, 36, 38), three the right DLPFC (31, 34, 37), and three bilateral DLPFC (26, 30, 33); two studies targeted non-DLPFC parietal regions. Only one trial combined rTMS with Tai Chi, whereas the remaining trials evaluated rTMS as a stand-alone intervention (34). With respect to stimulation intensity, three studies used 80% of the resting motor threshold (RMT), one used < 80% RMT, and the remainder applied ≥80% RMT. Detailed characteristics of the included studies are presented in Table 1. Baseline affective scores for studies contributing to the depression and anxiety analyses are additionally summarized in Supplementary Table 3.

### 3.3 Risk of bias

**Figure 2:**
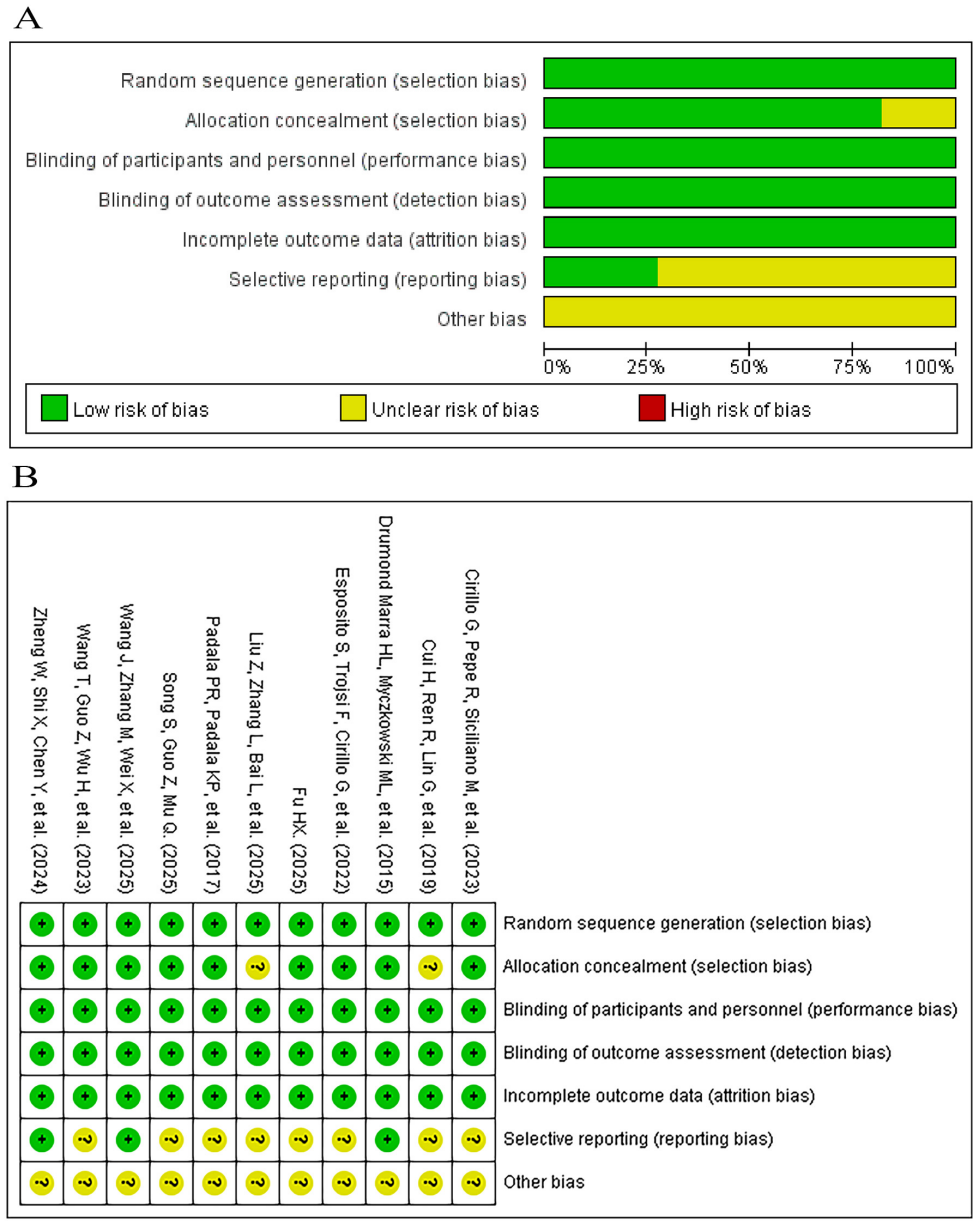
Summary and Visualization of Risk of Bias Assessment. **(A)** Deviation chart for risk of bias; **(B)** Risk of bias summary. “+” means low-risk bias; “?” means unclear risk bias; “–” means high-risk bias.

Methodological quality of the 11 RCTs was assessed using the Cochrane Risk of Bias tool. Two reviewers independently evaluated each study according to Cochrane standards (Figure 2). Overall, most studies were judged as having a low or unclear risk of bias across domains. Two studies provided insufficient information on sequence generation and/or allocation concealment, resulting in unclear risk for selection bias.

In addition, the selective reporting domain was re-evaluated by examining trial registration records, published protocols, and, when available, statistical analysis plans. Several studies were judged to have unclear risk in this domain because pre-registration or public protocol information was unavailable, and selective reporting could not be confidently ruled out. Detailed information on trial registration and protocol availability is provided in Supplementary Table 4.

### 3.4 Synthesis of results

To improve transparency and readability, Supplementary Table 5 lists the trials contributing to each pre-specified outcome and links them to the corresponding forest plots. We report pooled effects by cognitive domain, with outcome-specific contributing trials detailed in Supplementary Table 5.

#### 3.4.1 Global cognition

**Figure 3:**
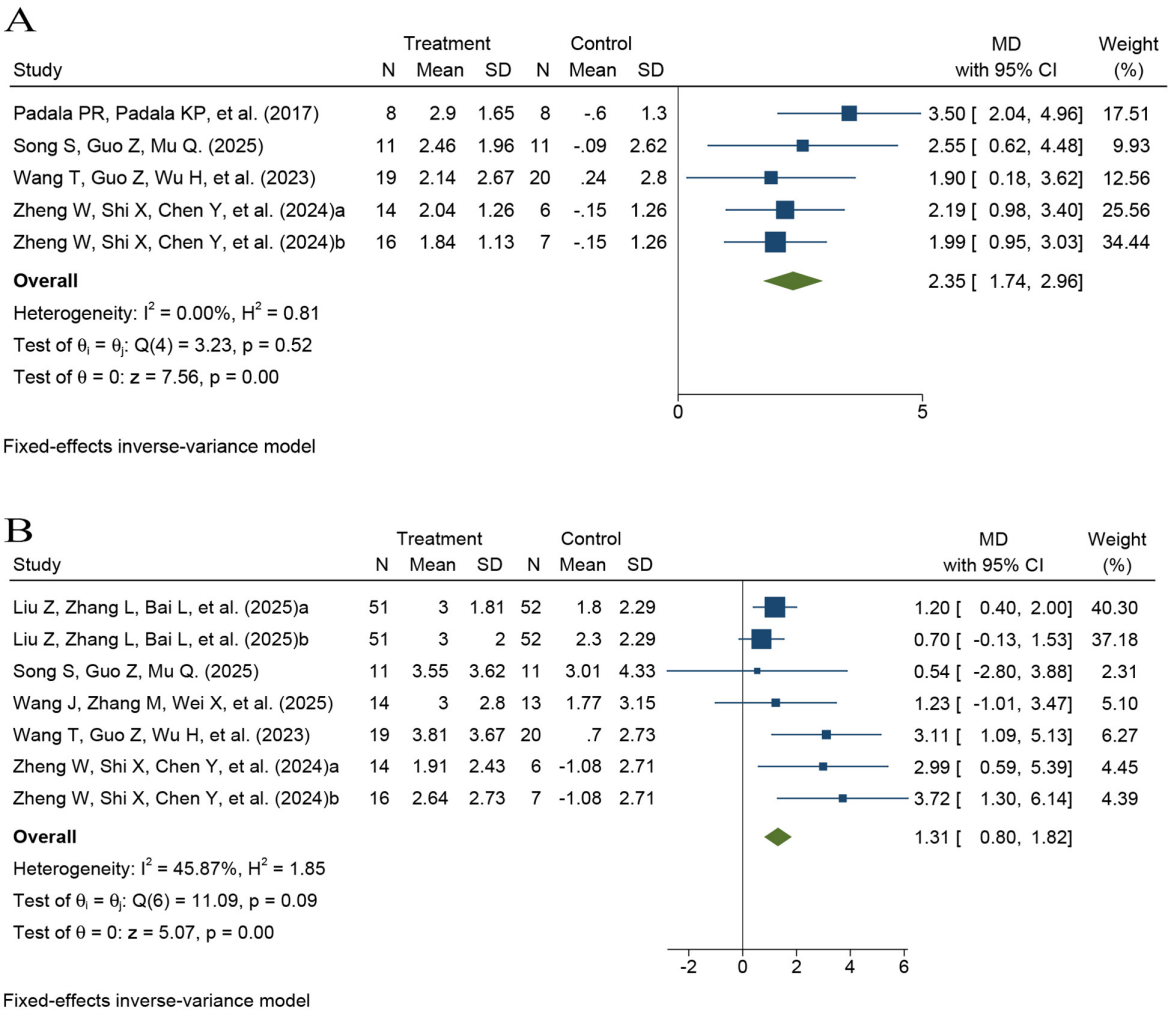
Forest plot showing the effects of rTMS on global cognitive outcomes. **(A)** MMSE; **(B)** MOCA.

In terms of overall cognitive function, five comparisons using the MMSE were included in the meta-analysis (*k* = 5; *n* = 120), including one three-arm trial that provided two comparisons (Supplementary Table 5) (35, 36, 38, 39). The pooled results using a fixed-effect model showed that the MMSE score in the rTMS group was significantly higher than that in the control group (*MD* = 2.35, 95% CI 1.74–2.96, *P* < 0.0001), with low heterogeneity (*I*^2^ = 0.00%; Figure 3A).

Additionally, six comparisons using the MoCA were included (*k* = 6; *n* = 337), including one three-arm trial that provided two comparisons (Supplementary Table 5) (34, 36–39). The pooled results using a fixed-effect model also indicated that rTMS significantly improved MoCA scores (*MD* = 1.31, 95% CI 0.80–1.82, *P* < 0.0001), with moderate heterogeneity (*I*^2^ = 45.87%; Figure 3B).

#### 3.4.2 Attention and executive functioning

**Figure 4:**
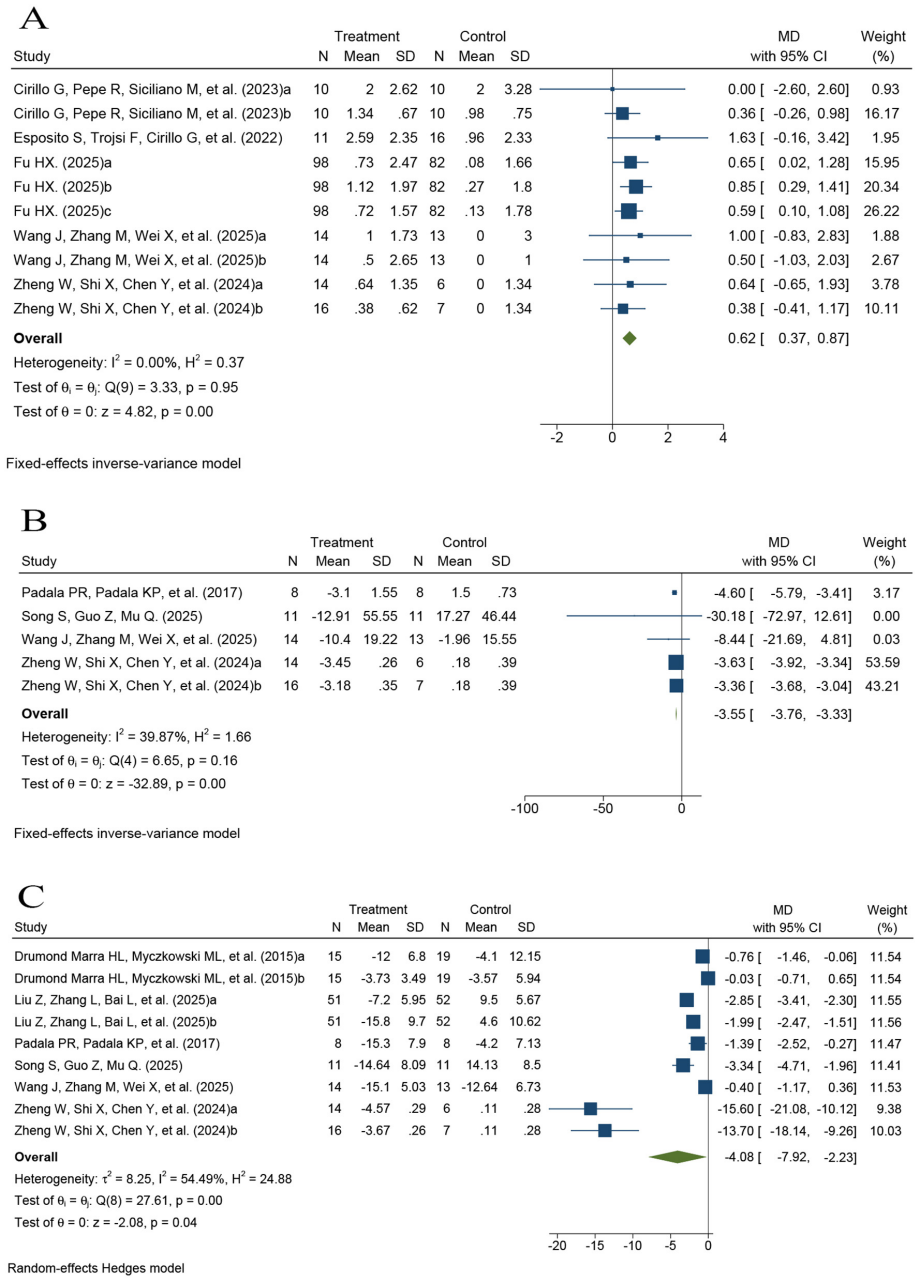
Forest plots of the effects of rTMS on attention and executive function. **(A)** Digit span test (DST); **(B)** Trail making test part A (TMT-A); and **(C)** Trail making test part B (TMT-B).

Six comparisons assessed the effects of rTMS on attention in patients with MCI using the DST (*k* = 6; *n* = 297), including one three-arm trial that provided two comparisons (Supplementary Table 5) (26, 30, 33, 37, 39). The pooled analysis using a fixed-effect model revealed a modest improvement in attention, as measured by the DST, in the rTMS group compared with controls (*MD* = 0.62, 95% CI 0.37–0.87, *P* < 0.001; *I*^2^ = 0.00%; Figure 4A).

Similarly, five comparisons evaluated attention and processing speed using the TMT-A (*k* = 5; *n* = 108), including one three-arm trial that provided two comparisons (Supplementary Table 5) (35–37, 39). Because TMT-A is a completion-time measure, with shorter times indicating better performance, pooled effects were synthesized using a fixed-effect model. The pooled results showed significantly shorter completion times in the rTMS group (*MD* = −3.55, 95% CI −3.76 to −3.33, *P* < 0.001; *I*^2^ = 39.87%; Figure 4B).

Seven comparisons evaluated the effects of rTMS on executive function in patients with MCI using the TMT-B (*k* = 7; *n* = 245), including one three-arm trial that provided two comparisons (Supplementary Table 5) (32, 34–37, 39). Because TMT-B is also a completion-time measure, with shorter times indicating better performance, and substantial heterogeneity was observed across studies (*I*^2^ = 54.49%), pooled effects were synthesized using a random-effects model. The pooled results indicated significantly shorter completion times in the rTMS group compared with controls (*MD* = −4.08, 95% CI −7.92 −2.23, *P* = 0.04), suggesting improved executive function (Figure 4C).

#### 3.4.3 Verbal memory

**Figure 5:**
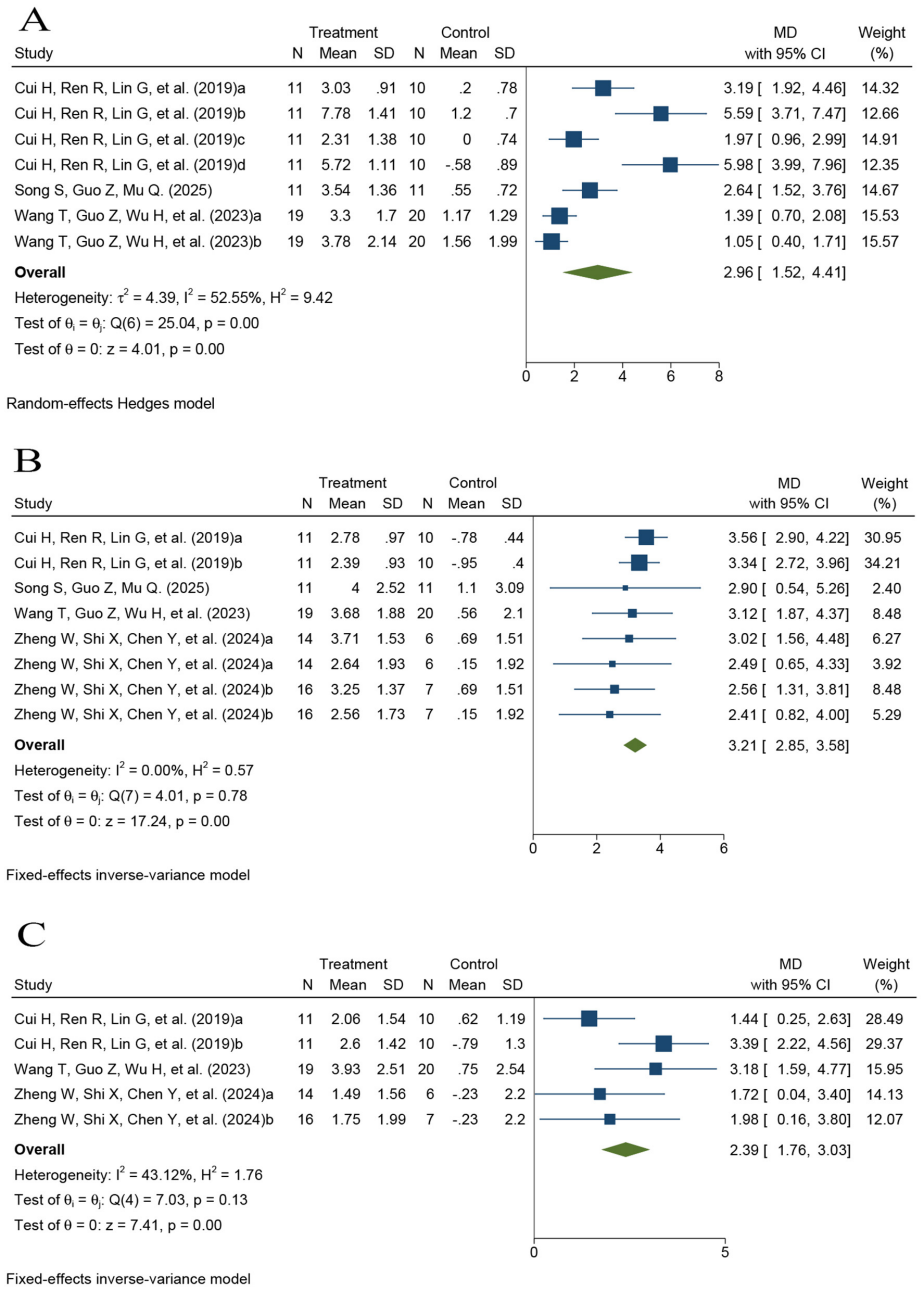
Forest plots of the effects of rTMS on verbal memory. **(A)** AVLT immediate recall; **(B)** AVLT delayed recall; **(C)** AVLT recognition.

Verbal memory was primarily assessed using the AVLT (immediate recall, delayed recall, and recognition). Three studies contributed to the AVLT immediate recall meta-analysis (*k* = 3; *n* = 111; see Supplementary Table 5 for contributing trials) (31, 36, 37). Because substantial heterogeneity was observed across studies, pooled effects were synthesized using a random-effects model. Pooled analysis demonstrated a significant improvement in immediate verbal memory in the rTMS group compared with controls (*MD* = 2.96, 95% CI 1.52–4.41, *P* < 0.001), with moderate heterogeneity across studies (*I*^2^ = 52.55%; Figure 5A).

Five comparisons contributed to the AVLT delayed recall analysis (*k* = 5; *n* = 125; see Supplementary Table 5 for contributing trials) (31, 36, 37, 39). The pooled results using a fixed-effect model showed a significant improvement in delayed verbal memory in the rTMS group compared with controls (*MD* = 3.21, 95% CI 2.85–3.58, *P* < 0.001), with no detectable heterogeneity (*I*^2^ = 0.00%; Figure 5B).

Four comparisons contributed to the AVLT recognition analysis (*k* = 4; *n* = 103), including one three-arm trial that provided two comparisons (Supplementary Table 5) (31, 37, 39). Pooled results using a fixed-effect model demonstrated significantly higher recognition scores in the rTMS group than in controls (*MD* = 2.39, 95% CI 1.76–3.03, *P* < 0.001), with low-to-moderate heterogeneity (*I*^2^ = 43.12%; Figure 5C).

#### 3.4.4 Language function

**Figure 6:**
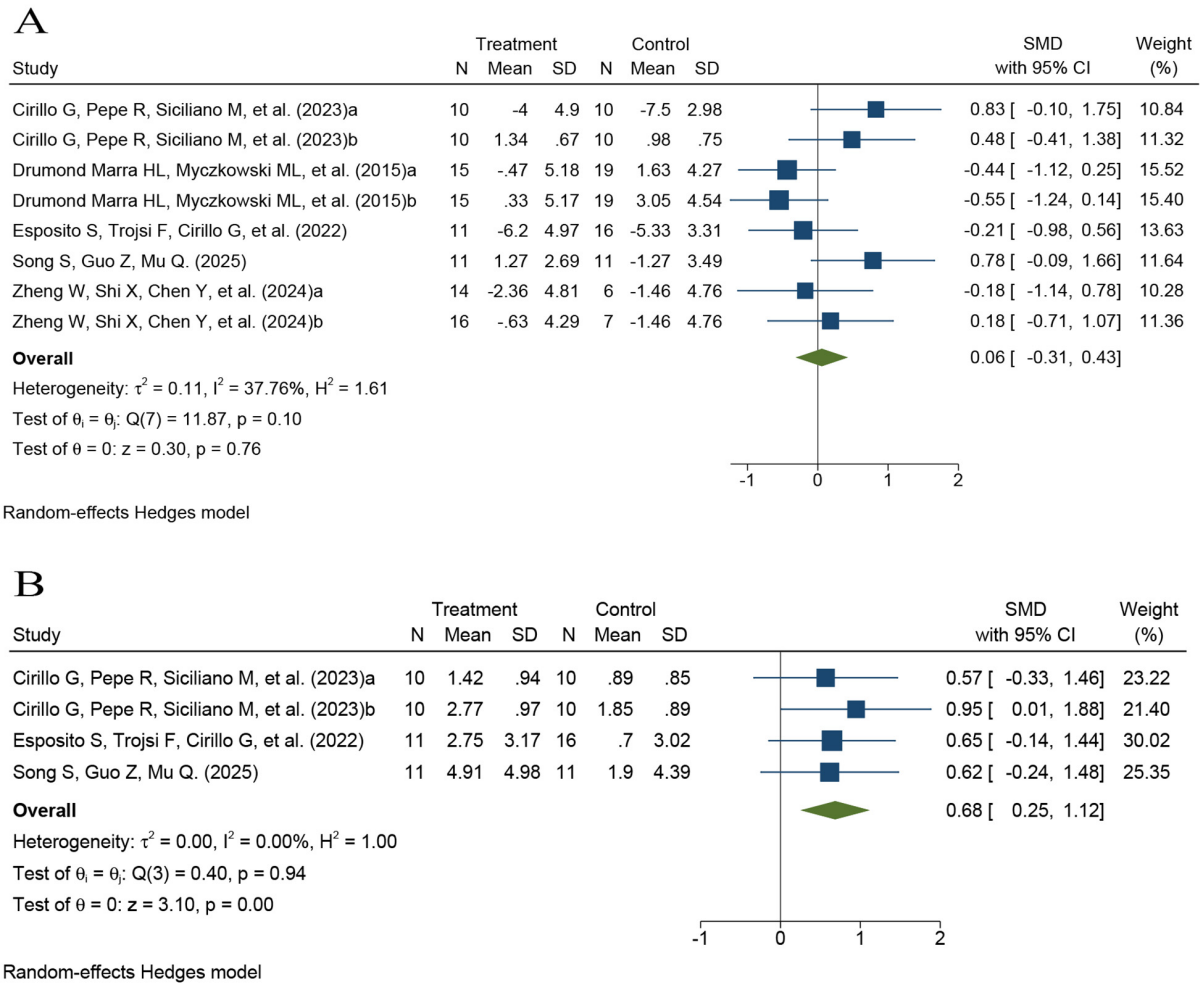
Forest plots of the effects of rTMS on language function. **(A)** Language fluency; **(B)** Naming performance. For outcomes synthesized using standardized mean differences, effect sizes were calculated as Hedges'g.

Language function was assessed using semantic fluency tasks, including the AFT, and naming tasks such as the Boston Naming Test or picture-naming measures. Six comparisons contributed to the semantic fluency analysis (*k* = 6; *n* = 134), including one three-arm trial that provided two comparisons (Supplementary Table 5) (30, 32, 33, 36, 39). Because semantic fluency was assessed using different measures across studies, pooled effects were synthesized using standardized mean differences calculated as Hedges'g and a random-effects model. The pooled analysis showed no significant improvement in language fluency in the rTMS group compared with controls (SMD = 0.06, 95% CI −0.31 to 0.43, *P* > 0.05), with low-to-moderate heterogeneity (*I*^2^ = 37.76%; Figure 6A).

Three studies contributed to the naming ability analysis (*k* = 3; *n* = 69; see Supplementary Table 5 for contributing trials) (30, 33, 36). Because naming ability was assessed using different instruments across studies, pooled effects were synthesized using standardized mean differences calculated as Hedges'g and a random-effects model. Pooled results demonstrated a significant improvement in naming performance in the rTMS group relative to controls (SMD = 0.68, 95% CI 0.25–1.12, *P* < 0.001), with no observed heterogeneity (*I*^2^ = 0.00%; Figure 6B).

#### 3.4.5 Mood symptoms

Affective outcomes were assessed using the Beck Anxiety Inventory (BAI) and the Hamilton Anxiety Rating Scale (HAMA) for anxiety. Depression outcomes were pooled across both self-reported and clinician-rated measures, including the Beck Depression Inventory-II (BDI-II) and the Hamilton Depression Rating Scale (HAMD).

Three studies contributed to the depression analysis (*k* = 3; *n* = 150; see Supplementary Table 5 for contributing trials) (30, 33, 34). Because depressive symptoms were assessed using different instruments across studies, including both self-reported and clinician-rated measures, pooled effects were synthesized using standardized mean differences calculated as Hedges'g and a random-effects model. The analysis showed a significant reduction in depression severity in the rTMS group compared with the control group (SMD = −0.79, 95% CI −1.19 to −0.39, *P* < 0.001), with moderate heterogeneity (*I*^2^ = 51.56%; Supplementary Figure 2A). Baseline depression scores for the contributing studies are summarized in Supplementary Table 3, and baseline depressive severity appeared to vary across studies.

Three studies also contributed to the anxiety analysis (*k* = 3; *n* = 150; see Supplementary Table 5 for contributing trials) (30, 33, 34). Because anxiety was also assessed using different instruments across studies, pooled effects were synthesized using standardized mean differences calculated as Hedges'g and a random-effects model. The pooled results demonstrated a significant improvement in anxiety in the rTMS group relative to the control group (SMD = −0.48, 95% CI −0.72 to −0.24, *P* < 0.001), with no observed heterogeneity (*I*^2^ = 0.00%; Supplementary Figure 2B).

### 3.5 Sensitivity analyses

Leave-one-out sensitivity analyses across all outcomes showed that the pooled estimates were stable. For global cognition, exclusion of two studies using image-based assessments produced results consistent with the main analysis, with significant improvements in MMSE (*MD* = 1.95, 95% CI 1.26–2.67) and MoCA (*MD* = 1.26, 95% CI 0.83–1.52). Overall, the findings indicate that the results were robust to variations in study inclusion and assessment methods.

### 3.6 Grade evaluation of evidence

The GRADE assessments and summary of evidence are presented in Table 2, with detailed domain-level evaluations provided in the raw data. Certainty of evidence varied across outcomes. Most outcomes were rated as moderate, including MMSE, MoCA, DST, TMT-A, AVLT delayed recall, AVLT recognition, naming ability, and anxiety. TMT-B, AVLT immediate recall, and depression were rated as low, while verbal fluency was rated as very low. Downgrading was mainly due to imprecision related to small sample sizes and, for some outcomes, inconsistency or wide confidence intervals.

**Table 2:** Summary of primary outcomes with GRADE evidence assessment.

Outcomes	No. of studies	No. of patients	Mean difference [±95% CI]	Anticipated absolute effects (95% CI)	*P*-value	Heterogeneity assessment	Reason for downgrading
			Grade evaluation	Risk with TMS		I^2^ [*p*-value]	
MMSE	5	120	*MD* = 2.35	1.37 higher	*P* < 0.001	*I*^2^ = 0.00%	Downgraded for imprecision due to the small total sample size (< 400 participants)
			95% CI [1.74 to 2.96] ⊕⊕⊕⊖ Moderate	(3.74 to 2.96 higher)		[*P* = 0.52]	
MOCA	6	337	*MD* = 1.31	0.80 higher	*P* < 0.001	*I*^2^ = 45.87%	Downgraded for imprecision due to the small total sample size (< 400 participants)
			95% CI [0.80 to 1.82] ⊕⊕⊕⊖ Moderate	(0.80 to 1.82 higher)		[*P* = 0.09]	
DST	6	297	*MD* = 0.62	0.37 higher	*P* < 0.001	*I*^2^ = 0.00%	Downgraded for imprecision due to the small total sample size (< 400 participants)
			95% CI [0.37 to 0.87] ⊕⊕⊕⊖ Moderate	(0.37 to 0.87 higher)		[*P* = 0.95]	
TMT-A	5	108	*MD* = −3.55	3.76 lower	*P* < 0.001	*I*^2^ = 39.87%	Downgraded for imprecision due to the small total sample size (< 400 participants)
			95% CI [−3.76 to −3.33] ⊕⊕⊕⊖ Moderate	(3.76 to 3.33 lower)		[*P* = 0.16]	
TMT B	7	245	*MD*=-4.08	7.92 lower	*P* = 0.04	*I*^2^ = 54.49%	Downgraded for imprecision due to the small total sample size (< 400 participants) and high inconsistency (*I*^2^ > 50%)
			95% CI [−7.92 to −2.23] ⊕⊕⊖⊖ Low	(7.92 to 2.23 lower)		[*P* < 0.001]	
AVLT-immediate recall	3	111	*MD* = 2.96	2.97 higher	*P* < 0.001	*I*^2^ = 52.55%	Downgraded for imprecision due to the small total sample size (< 400 participants) and high inconsistency (*I*^2^ > 50%)
			95% CI [1.52 to 4.41] ⊕⊕⊖⊖ Low	(1.52 to 4.41 higher)		[*P* < 0.001]	
AVLT-delayed recall	5	125	*MD* = 3.21	2.85 higher	*P* < 0.001	*I*^2^ = 0.00%	Downgraded for imprecision due to the small total sample size (< 400 participants)
			95% CI [2.85 to 3.58] ⊕⊕⊕⊖ Moderate	(2.85 to 3.58 higher)		[*P* = 0.78]	
AVLT-recognition recall	4	103	*MD* = 2.39	1.76 higher	*P* < 0.001	*I*^2^ = 43.12%	Downgraded for imprecision due to the small total sample size (< 400 participants)
			95% CI [1.76 to 3.03] ⊕⊕⊖⊖ Moderate	(1.76 to 3.03 higher)		[*P* = 0.13]	
Verbal fluency test	6	134	*SMD* = 0.06	0.31 higher	*P* = 0.76	*I*^2^ = 37.76%	Downgraded for imprecision due to the small total sample size (< 400 participants) and wide confidence intervals crossing the line of no effect
			95% CI [−0.31 to 0.43] ⊕⊖⊖⊖ Very low	(−0.31 to 0.43 higher)		[*P* = 0.10]	
Naming ability	3	69	*SMD* = 0.68	0.25 higher	*P* < 0.001	*I*^2^ = 0.00%	Downgraded for imprecision due to the small total sample size (< 400 participants)
			95% CI [0.25 to 1.12] ⊕⊕⊕⊖ Moderate	(0.25 to 1.12 higher)		[*P* = 0.94]	
Depression	3	150	*SMD* = −0.79	1.19 lower	*P* < 0.001	*I*^2^ = 51.56%	Downgraded for imprecision due to the small total sample size (< 400 participants) and high inconsistency (*I*^2^ > 50%)
			95% CI [−1.19 to −0.39] ⊕⊕⊖⊖ Low	(1.19 to 0.39 lower)		[*P* = 0.09]	
Anxiety	3	150	*SMD* = −0.48	0.72 lower	*P* < 0.001	*I*^2^ = 0.00%	Downgraded for imprecision due to the small total sample size (< 400 participants)
			95% CI [−0.72 to −0.24] ⊕⊕⊕⊖ Moderate	(0.72 to 0.24 lower)		[*P* = 0.42]	

TMS, transcranial magnetic stimulation; RCT, randomized controlled trial; MMSE, mini-mental state examination; MOCA, montreal cognitive assessment; AVLT, auditory verbal learning test; TMT, trail making test; DST, digit span test.

### 3.7 Adverse events

Among the 11 studies included in this meta-analysis, 7 reported adverse events. One study reported no adverse events, while the remaining six studies described mild adverse reactions (26, 30, 32, 35, 37–39). Reported events were generally mild and transient, most commonly including stimulation-site discomfort or headache, transient dizziness, and facial twitching, all of which were well tolerated and resolved with rest. Some studies also reported mild nausea, vomiting, diarrhea, shock sensations, or insomnia, none of which were severe. Overall, no serious adverse events were reported. However, adverse event reporting was incomplete in several studies, which may limit a comprehensive evaluation of the safety of rTMS. Larger, well-designed RCTs with standardized safety reporting are therefore needed to confirm further both the efficacy and safety of rTMS in MCI.

## 4 Discussion

This systematic review and meta-analysis synthesized RCTs in MCI-only samples to characterize the domain-specific cognitive and affective effects and safety of rTMS. Overall, rTMS was associated with statistically significant improvements in global cognition as assessed by MMSE and MoCA. In studies that administered the AVLT, rTMS showed more pronounced benefits in verbal memory, particularly delayed recall and recognition, suggesting a potential influence on memory consolidation. Benefits were also observed for selected outcomes in attention, executive function, and naming ability, whereas language fluency showed no significant improvement. Depressive and anxiety symptoms were reduced, consistent with the modulation of pre-frontal circuits involved in both cognition and affect. rTMS was generally well tolerated, with only mild and transient adverse events reported. We enhanced transparency by reporting the contributing trials and sample sizes for each outcome and assessed robustness and certainty of evidence using sensitivity analyses and GRADE. However, small samples, protocol variability, and inconsistent reporting of stimulation characteristics limited parameter-focused inference; thus, current evidence does not establish an “optimal” stimulation regimen and should be interpreted primarily as evidence of domain-level effects.

Compared with prior reviews, our work extends the evidence base in two important ways. First, by restricting eligibility to MCI-only RCTs, we reduced diagnostic heterogeneity introduced by pooling MCI with early AD/dementia spectra, thereby improving the clinical interpretability of pooled effects specifically for the prodromal stage. Second, rather than focusing primarily on global cognitive scales, we synthesized domain-specific outcomes, which helps clarify differential responsiveness across cognitive functions and aligns with our *a priori* hypothesis that rTMS targeting pre-frontal control networks would preferentially benefit executive/attention processes. In this context, the present findings suggest that rTMS may improve global cognitive function in patients with MCI, as reflected by improvements in both MMSE and MoCA scores. Because global cognitive scales encompass multiple domains (memory, attention, executive function, and language), these gains may reflect broad but modest improvements rather than a marked change in a single function. Our results are broadly consistent with prior RCTs and systematic reviews reporting beneficial effects of pre-frontal rTMS in MCI, particularly among individuals with milder baseline impairment or longer intervention durations (27, 37, 40). From a clinical perspective, the observed improvements may be meaningful: minimal clinically important differences (MCIDs) have been suggested to be approximately 1–3 points for the MMSE and 1–2 points for the MoCA (41, 42), and the magnitude of improvement in our analysis approached or reached these thresholds. Sensitivity analyses supported the robustness of these findings. Mechanistically, rTMS may modulate cortical excitability and influence distributed networks beyond the stimulation site (43, 44). Although optimal parameters remain to be established, most included trials applied high-frequency stimulation to the DLPFC, a region central to working memory and cognitive control (45), which is consistent with the observed benefits in global cognition and selected domain-specific measures (46, 47).

Regarding memory outcomes, this study found that rTMS was associated with relatively consistent and stable improvements in verbal memory, particularly in AVLT delayed recall and recognition. Delayed recall reflects memory consolidation and retention, while recognition demonstrates the preservation of memory traces; both measures are commonly used in clinical practice to distinguish MCI from normal aging and are closely related to longitudinal cognitive trajectories (48, 49). The low between-study heterogeneity observed for these outcomes indicates good consistency across studies. Although no established MCID has been defined for AVLT delayed recall or recognition, prior research suggests that even modest but sustained improvements in these memory measures may be clinically meaningful in the MCI stage, given their strong association with disease progression (50, 51). Previous studies have also reported beneficial effects of rTMS, particularly high-frequency stimulation targeting the DLPFC, on memory-related cognitive functions (52, 53). The DLPFC, especially in the left hemisphere, is a key node within language and memory networks and maintains extensive connections with hippocampal and other cortical–subcortical regions. Neuroimaging studies using diffusion tensor imaging and functional MRI have linked cognitive performance to white-matter integrity and functional activation within these networks, and longitudinal evidence suggests that longer courses of rTMS may be associated with attenuated structural decline in stimulation-related regions (54). Taken together, the stable memory improvements observed in this meta-analysis are broadly consistent with existing evidence on rTMS-related modulation of pre-frontal cognitive networks; however, as a meta-analysis, the present study cannot directly verify underlying neural mechanisms, which require further investigation in dedicated imaging and mechanistic studies.

This study further showed that rTMS has domain-specific effects on executive function, language, and affective outcomes. Improvements in executive function, as measured by TMT-B, were observed but were less stable than those seen for memory, suggesting greater sensitivity to stimulation parameters and individual variability. In the language domain, rTMS was associated with improved naming ability, whereas no significant effect was found for language fluency, indicating differential responsiveness across language subdomains. In addition, reductions in depressive and anxiety symptoms were observed. Given the high prevalence of affective disturbances in MCI, these changes may be clinically relevant and could indirectly support cognitive performance by improving motivation, attention, and treatment engagement. Overall, these findings highlight the heterogeneous effects of rTMS across cognitive and emotional domains. From an explanatory standpoint, executive and language functions are strongly left-lateralized (55). Neuroimaging studies have demonstrated asymmetric frontal white-matter connectivity, with greater integrity in the left frontal lobe associated with better cognitive performance, while MCI is characterized by reduced left frontal white-matter integrity (56, 57). Disruption of this hemispheric asymmetry may therefore limit responsiveness in certain cognitive domains (58). Furthermore, language fluency relies on more distributed neural networks, whereas naming depends on more localized lexical–semantic processing, which may account for their differing sensitivity to focal pre-frontal stimulation (59, 60). The effects of repetitive rTMS may also extend beyond the stimulation site through anatomically and functionally connected networks, including interhemispheric pathways, further contributing to domain-specific treatment responses.

Notably, the pooled effect on depressive symptoms was among the largest observed in this review and appeared potentially clinically meaningful. This result may be partly related to the stimulation targets used in the included studies, as the dorsolateral pre-frontal cortex was the pre-dominant target in most trials. Given the central role of the DLPFC in both mood regulation and cognitive control, a relatively strong effect on depressive symptoms is mechanistically plausible. This pattern may also reflect differential responsiveness across outcome domains, as affective symptoms may be more directly influenced by pre-frontal stimulation than cognitive functions that rely on broader and more distributed neural networks (61). At the same time, baseline depressive severity appeared to vary across the contributing studies rather than being uniformly moderate or severe. Specifically, the two studies using the BDI-II suggested mild depressive symptom burden at baseline, whereas the study using the HAMD indicated generally low baseline depression scores. This suggests that the relatively large pooled effect cannot be explained solely by high baseline depression severity. Because depressive symptoms can adversely affect motivation, attention, and cognitive test performance, part of the observed cognitive benefit may have been mediated by mood improvement rather than direct modulation of cognitive networks alone (62). In addition, baseline depressive burden may also have moderated treatment responsiveness, but the currently available study-level data do not allow formal testing of moderation or mediation pathways. Therefore, affective improvement should still be considered an important potential source of confounding when interpreting the cognitive outcomes. Moreover, because depression outcomes were synthesized across both self-reported and clinician-rated instruments using SMDs, the pooled magnitude should be interpreted with some caution in terms of direct clinical comparability.

Across the included RCTs, stimulation prescriptions and active arms varied in pattern (HF-rTMS, LF-rTMS, and iTBS), target site, intensity, and delivered dose. Most protocols targeted the DLPFC and commonly used 10 Hz, but 1 Hz, 20 Hz, and iTBS were also applied, with intensities roughly 70%−120% RMT/MT and treatment courses of 10–40 sessions. For comparability, we summarized exposure as cumulative dose (total pulses per course) in Table 1, using reported values or calculating totals from pulses/session and total sessions when possible. Stimulation was delivered alone in all but one trial, which combined rTMS with Tai Chi and may introduce co-intervention effects. rTMS appeared generally tolerable in MCI: adverse events were mostly mild and transient, with no serious events reported, although reporting was incomplete in some studies. The GRADE assessment further suggested that certainty was not uniform across outcomes. Evidence for global cognition and some AVLT-based verbal memory measures was relatively more consistent and was generally rated as moderate, whereas evidence for TMT-B, AVLT immediate recall, and depression was rated as low, and verbal fluency was rated as very low. These differences indicate that not all observed effects should be interpreted with the same level of confidence. The marked variation in effect size across outcomes may reflect domain-specific responsiveness to rTMS, differences in the sensitivity and construct coverage of assessment tools, and between-study heterogeneity in stimulation protocols. Another important priority for future research is to determine which stimulation protocols are most effective for MCI. Given the limited number of available trials and the substantial variation in stimulation pattern, frequency, intensity, cumulative pulse dose, and treatment duration, current evidence is still insufficient to identify an optimal protocol. Future studies should therefore more systematically compare stimulation parameters and may also benefit from insights from other TMS fields with larger and more mature evidence bases. Accordingly, the more consistent findings for global cognition and selected AVLT-based verbal memory outcomes may be interpreted with relatively greater confidence, whereas positive findings for some executive, affective, and language measures should be viewed more cautiously because they were supported by smaller evidence bases and lower certainty ratings. In addition, the evidence base was geographically concentrated, with most included studies conducted in China, while the remaining studies from other countries were relatively small. As a result, patients from other healthcare systems and ethnic backgrounds were underrepresented, which may limit the generalizability of the present findings. Overall, rTMS may benefit cognition and affect in MCI, but larger trials with longer follow-up, more standardized reporting of pulse-dose metrics and adverse events, and more direct comparisons of stimulation protocols are needed.

More broadly, the present findings underscore the importance of prospective study pre-registration in this field. By specifying hypotheses, primary outcomes, and analysis plans before data collection, pre-registration can reduce selective reporting and improve the transparency of confirmatory vs. exploratory analyses (63). Given the relative maturity of rTMS research and the increasingly explicit hypotheses being tested in MCI, Registered Reports may provide an even stronger framework for future trials. This may be particularly relevant in this field, where studies often test pre-specified stimulation targets, frequencies, and cognitively defined outcome domains. Because the Registered Reports format includes peer review and in-principle acceptance before results are known, it may help reduce publication bias and other forms of outcome-driven bias while strengthening methodological and analytical rigor (64). Beyond improving methodological rigor and reporting transparency, another relevant future direction is to explore more integrative intervention strategies. Another potentially relevant future direction is to explore whether brain stimulation approaches such as rTMS can be combined with self-regulation-based interventions such as neurofeedback (65). Neurofeedback has also shown potential in MCI and dementia-related populations, although current evidence remains limited and heterogeneous (66). A combined approach may be of interest because rTMS and neurofeedback could act through partially complementary mechanisms, with the former modulating cortical excitability and network plasticity and the latter promoting endogenous self-regulation of brain activity. In addition, future studies may explore closed-loop stimulation paradigms that combine rTMS with simultaneous EEG- or fNIRS-based monitoring, in which stimulation is triggered when pre-defined neurophysiological thresholds or brain-state markers are reached. This strategy may be particularly relevant for DLPFC-targeted neuromodulation, as it could improve state-dependent targeting and the precision of stimulation delivery (67). Future studies could therefore examine whether such multimodal and adaptive strategies improve the magnitude or durability of cognitive benefits.

## 5 Limitations

Several limitations should be noted. Although we restricted inclusion to RCTs, several outcome-specific analyses were based on small samples, leading to imprecise estimates and constituting the primary reason for downgrading certainty in the GRADE assessment. In addition, small studies may be more susceptible to publication bias, selective outcome reporting, and other reporting-related biases, which can contribute to overestimation of treatment effects. Although standardized mean differences were calculated as Hedges'g to reduce small-sample bias in effect size estimation, this approach cannot correct for these broader sources of bias (68). Therefore, the pooled effects, particularly for outcomes supported by relatively small evidence bases, should be interpreted cautiously.

Stimulation prescriptions varied across trials (e.g., target site, pattern/frequency, intensity, course length, and cumulative pulse dose), which may have influenced pooled effects; however, statistical heterogeneity was generally low to moderate and not uniform across outcomes. Differences in cognitive assessment instruments, together with figure-based data extraction for some studies, could have introduced measurement error, although sensitivity analyses supported the robustness of key findings. Trial registration or publicly available protocol information was limited for several included studies, which introduced uncertainty in the assessment of selective reporting bias. Most included studies were conducted in China, whereas the non-Chinese studies were relatively small, limiting the geographic and ethnic representativeness of the evidence base and reducing the generalizability of the findings. Baseline affective burden was variably reported across studies, and only a small number of trials contributed to the affective analyses. Because no individual participant data were available, we could not formally test whether depressive symptoms moderated or mediated the cognitive outcomes; therefore, mood improvement remains a potential source of confounding when interpreting the observed cognitive benefits. Adverse events were also incompletely and inconsistently reported, limiting the safety appraisal. One trial combined stimulation with Tai Chi, which may have introduced co-intervention effects and complicated attribution to rTMS alone. Finally, follow-up durations were typically short, precluding conclusions regarding sustained benefits or effects on longer-term cognitive trajectories and progression to dementia.

## 6 Conclusion

This MCI-only, domain-specific meta-analysis indicates that rTMS improves global cognition (MMSE/MoCA) and yields more consistent benefits for AVLT-based verbal memory (delayed recall/recognition). Favorable effects were also observed in selected executive/attention measures and naming, with additional improvements seen in depressive and anxiety symptoms. rTMS was generally well tolerated, with mostly mild and transient adverse events. Given the mostly low-to-moderate certainty and protocol heterogeneity, larger high-quality RCTs with longer follow-up are needed.

## Data Availability

The original contributions presented in the study are included in the article/Supplementary material, further inquiries can be directed to the corresponding authors.
