# Supplementary material for: Efficacy of transcranial magnetic stimulation for mild cognitive impairment: a systematic review and meta-analysis of randomized controlled trials
Source: Front Neurol. 2026 May 18;17:1788223. doi: 10.3389/fneur.2026.1788223 (PMC13222799; doi:10.3389/fneur.2026.1788223)
Supplement: Supplementary file 1 [file Table_1.docx]

**Supplementary Table 1.** Search strategies.

#1 Dementia **[Mesh]**

#2 Delirium **[Mesh]**

#3 Alzheimer Disease **[Mesh]**

#4 Cognition Disorders **[Mesh]**

**#**5 Neurocognitive Disorders **[Mesh]**

**#**6 Cognitive Dysfunction **[Mesh]**

#7 (Dementia **OR** Delirium **OR** Amnestic **OR** MCI **OR** "Cognitive Disorders" **OR** "Mild Cognitive Impairment" OR "Cognitive Dysfunction") **[Title/Abstract]**

#8 ((Memory **OR** "Executive Function Disorders") **And** Disorders) **[Title/Abstract]**

#9 (dement* **OR** alzheimer* **OR** deliri* OR "cognit* impair*") **[Title/Abstract]**

#10 ((cognit* **OR** memory **OR** cerebr* **OR** mental*) **And** (declin* **OR** impair* **OR** los* **OR** deteriorat* **OR** degenerat* **OR** complain* **OR** disturb* **OR** disorder*)) **[Title/Abstract]**

#11 ("preclinical AD" **OR** "pre‐clinical AD") **[Title/Abstract]**

#12 ("preclinical alzheimer*" **OR** "pre‐clinical alzheimer*") **[Title/Abstract]**

#13 (aMCI **OR** MCIa) **[Title/Abstract]**

#14 "mild neurocognit* disorder*" **[Title/Abstract]**

#15 #1 OR #2 OR #3 OR #4 OR #5 OR #6 OR #7 OR #8 OR #9 OR #10 OR #11 OR #12 OR #13 OR #14

#16 Transcranial Magnetic Stimulation **[MeSH]**

#17 (transcrani* **OR** magnetic **OR** TMS **OR** rTMS **OR** iTMS **OR** sTMS **OR** dTMS **OR** nTMS) **[Title/Abstract]**

#18 ((non‐invasive **OR** noninvasive) **and** brain stimulat*) **[Title/Abstract]**

#19 (theta‐burst* **OR** thetaburst* **OR** TBS **OR** iTBS **OR** aiTBS) **[Title/Abstract]**

#20 #15 OR #16 OR #17 OR #18

#21 (randomized controlled trial[pt] OR controlled clinical trial[pt] OR randomized[tiab] OR placebo[tiab] OR clinical trials as topic[mesh:noexp] OR randomly[tiab] OR trial[ti]) NOT (animals [mh] NOT (humans [mh] AND animals[mh]))

#21 #15 and #20 and #21
