# Supplementary material for: Efficacy of transcranial magnetic stimulation for mild cognitive impairment: a systematic review and meta-analysis of randomized controlled trials
Source: Front Neurol. 2026 May 18;17:1788223. doi: 10.3389/fneur.2026.1788223 (PMC13222799; doi:10.3389/fneur.2026.1788223)
Supplement: Supplementary file 2 [file Table_2.docx]

**Supplementary Table 2.** Full-text articles were excluded after eligibility assessment, and reasons (n = 14).

| **Excluded study (Author, Year)** | **Excluded full-text study (full citation)** | **Reason for exclusion** |
| --- | --- | --- |
| Aghamoosa, S., et al. (2025) | Aghamoosa S, et al. Accelerated iTBS-Induced changes in resting-state functional connectivity correspond with cognitive improvement in amnestic MCI. Brain Stimul. 2025;18(3):957-964. | Non-randomized design |
| Anderkova, L., et al. (2015) | Anderkova L, et al. Distinct Pattern of Gray Matter Atrophy in Mild Alzheimer's Disease Impacts on Cognitive Outcomes of Noninvasive Brain Stimulation. J Alzheimers Dis. 2015;48(1):251-260. |  |
| Anderkova, L., et al. (2016) | Anderkova L, et al. Grey matter atrophy in mild Alzheimer's disease impacts on cognitive effects of noninvasive brain stimulation. |  |
| Sacco L, et al. (2023) | Sacco L, et al. Transcranial Magnetic Stimulation Improves Executive Functioning through modulation of Social Cognitive networks in patients with mild cognitive impairment: preliminary results[J]. Diagnostics, 2023, 13(3): 415. |  |
| Cheng C P W, et al. (2018) | Cheng C P W, et al. Effects of repetitive transcranial magnetic stimulation on improvement of cognition in elderly patients with cognitive impairment: a systematic review and meta‐analysis[J]. International journal of geriatric psychiatry, 2018, 33(1): e1-e13. |  |
| Yang Linlin, et al. (2013) | Yang Linlin, et al. Research on Event-related Potentials in the Treatment of Mild Cognitive Impairment with High-frequency Repetitive Transcranial Magnetic Stimulation [C]// Chinese Medical Association, Chinese Society of Psychiatry. The 11th National Academic Conference of Psychiatry of the Chinese Medical Association and the 3rd Asian Conference on Neuro-psychopharmacology. 2013: 250. | Conference abstract format |
| Gong Jingyi, et al. (2024). | Gong Jingyi, et al. Research on Brain Activation during Speech Fluency Tasks in Patients with Mild Cognitive Impairment Using Near-Infrared Brain Functional Imaging Technology [C]//Shenzhen Rehabilitation Medical Association, Hong Kong Occupational Therapy Institute, Greater Bay Area Rehabilitation Medical Association, Chinese Rehabilitation Therapist Association, Chinese County-level Rehabilitation Alliance. 2024 Shenzhen International Rehabilitation Forum (21st) Outstanding Abstract Collection. |  |
| Han D, et al. (2025). | Han D, et al. Effects of Combined Transcranial Magnetic Stimulation and Photobiomodulation in Mild Cognitive Impairment: A Randomized Controlled Trial[J]. Journal of Biophotonics, 2025, 18(8): e70019. | Missing primary outcomes |
| Julkunen P, et al. (2008) | Julkunen P, et al. Navigated TMS combined with EEG in mild cognitive impairment and Alzheimer's disease: a pilot study[J]. Journal of neuroscience methods, 2008, 172(2): 270-276. |  |
| Taylor, J. L., et al. (2025). | Taylor J L, Bhatt P, Hernandez B, et al. Network-targeted transcranial magnetic stimulation (TMS) for mild cognitive impairment (MCI)[J]. NeuroImage: Clinical, 2025, 47: 103819. |  |
| Papallo, S., et al. (2024). | Papallo S, Di Nardo F, Siciliano M, et al. Functional connectome controllability in patients with mild cognitive impairment after repetitive transcranial magnetic stimulation of the dorsolateral prefrontal cortex[J]. Journal of Clinical Medicine, 2024, 13(18): 5367. |  |
| Liang, J., et al. (2025) | Liang J, et al. Individual electric field in cortical white matter is correlated with cognitive improvement in patients with mild cognitive impairment due to Alzheimer's disease after repetitive transcranial magnetic stimulation treatment[J]. Journal of Alzheimer’s Disease, 2025, 106(2): 703-715. | Unavailable full text |
| Zhang, et al. (2024) | Zhang Tianjiao, et al. Clinical Application of Transcranial Magnetic Stimulation Technology in the Treatment of Alzheimer's Disease and Mild Cognitive Impairment [J]. Chinese Journal of Rehabilitation Medicine, 2024, 39(07): 1060-1067. | non-English language |
| Xu, et al. (2025) | Xu Qingbin. The Impact of Repetitive Transcranial Magnetic Stimulation Therapy on Cerebral Blood Flow Circulation and Cognitive Function in Patients with Mild Cognitive Impairment after Stroke [J]. Journal of Practical Laboratory Medicine, 2025, 17(02): 106-109. |  |
