# Supplementary material for: Efficacy of transcranial magnetic stimulation for mild cognitive impairment: a systematic review and meta-analysis of randomized controlled trials
Source: Front Neurol. 2026 May 18;17:1788223. doi: 10.3389/fneur.2026.1788223 (PMC13222799; doi:10.3389/fneur.2026.1788223)
Supplement: Supplementary file 3 [file Table_3.docx]

**Supplementary Table 3.** Baseline affective symptom scores in studies contributing to the depression and anxiety analyses.

| **Study** | **Depression scale** | **Baseline depression score (intervention group)** | **Baseline depression score (control group)** |
| --- | --- | --- | --- |
| Cirillo G, Pepe R, Siciliano M, et al. (2023) | Beck Depression Inventory II scale (BDI-II) | 15.5 ± 14.44 | 12.00 ± 10.19 |
| Esposito S, Trojsi F, Cirillo G, et al. (2022) | Beck Depression Inventory II scale (BDI-II) | 16.77 ± 9.88 | 16.23 ± 10.19 |
| Liu Z, Zhang L, Bai L, et al. (2025) | Hamilton Depression Rating Scale | 5.60 ± 3.10 | 5.40 ± 2.50 |
| **Study** | **Anxiety scale** | **Baseline anxiety score (intervention group)** | **Baseline anxiety score (control group)** |
| Cirillo G, Pepe R, Siciliano M, et al. (2023) | Beck Anxiety Inventory (BAI) | 4.00 ± 10.19 | 8.50 ± 8.15 |
| Esposito S, Trojsi F, Cirillo G, et al. (2022) | Beck Anxiety Inventory (BAI) | 3.19 ± 2.12 | 3.32 ± 1.16 |
| Liu Z, Zhang L, Bai L, et al. (2025) | Hamilton Anxiety Rating Scale | 7.90 ± 3.20 | 7.50 ± 3.40 |

Note: Baseline affective scores are presented as mean ± standard deviation (SD) for the intervention and control groups in studies contributing to the depression and anxiety analyses.
