# Supplementary material for: Efficacy of transcranial magnetic stimulation for mild cognitive impairment: a systematic review and meta-analysis of randomized controlled trials
Source: Front Neurol. 2026 May 18;17:1788223. doi: 10.3389/fneur.2026.1788223 (PMC13222799; doi:10.3389/fneur.2026.1788223)
Supplement: Supplementary file 4 [file Table_4.docx]

**Supplementary Table 4.** Trial registration, protocol availability, and risk-of-bias judgments for selective reporting in the included studies.

| **Study** | Registration  reported | Registry/source | Registration ID | Protocol available | Prospectively registered/ retrospectively  registered/unclear | RoB 2 judgment  for the selection of  the reported result | Brief rationale |
| --- | --- | --- | --- | --- | --- | --- | --- |
|  |  |  |  |  |  |  |  |
| Cirillo G, Pepe R, Siciliano M, et al. (2023) | No | Not reported | Not reported | No | Unclear | Some concerns | No trial registration or public protocol identified;  selective reporting could not be ruled out. |
| Cui H, Ren R, Lin G, et al. (2019) | No | Not reported | Not reported | No | Unclear | Some concerns | No trial registration or public protocol identified;  selective reporting could not be ruled out. |
| Drumond Marra HL, Myczkowski ML, et al. (2015) | Yes | ClinicalTrials.gov | NCT01292382 | No / Not  publicly available | Unclear | Some concerns | Trial registration was reported, but no public protocol  or prespecified analysis plan was identified. |
| Esposito S, Trojsi F, Cirillo G, et al. (2022) | No | Not reported | Not reported | No | Unclear | Some concerns | No trial registration or public protocol identified;  selective reporting could not be ruled out. |
| Fu HX. (2025) | No | Not reported | Not reported | No | Unclear | Some concerns | No trial registration or public protocol identified;  selective reporting could not be ruled out. |
| Liu Z, Zhang L, Bai L, et al. (2025) | No | Supplement material | Not reported | Yes | Unclear | Some concerns | Trial protocol and statistical analysis plan were available,  but no trial registration information was reported. |
| Padala PR, Padala KP, et al. (2017) | No | Not reported | Not reported | No | Unclear | Some concerns | No trial registration or public protocol identified;  selective reporting could not be ruled out. |
| Song S, Guo Z, Mu Q. (2025) | No | Not reported | Not reported | No | Unclear | Some concerns | No trial registration or public protocol identified;  selective reporting could not be ruled out. |
| Wang J, Zhang M, Wei X, et al. (2025) | Yes | Chinese Clinical  Trial Registry | ChiCTR  1900021795 | No / Not  publicly available | Unclear | Some concerns | Trial registration was reported, but no public protocol or  prespecified analysis plan was identified. |
| Wang T, Guo Z, Wu H, et al. (2023) | No | Not reported | Not reported | No | Unclear | Some concerns | No trial registration or public protocol identified;  selective reporting could not be ruled out. |
| Zheng W, Shi X, Chen Y, et al. (2024) | Yes | Chinese Clinical  Trial Registry | ChiCTR  2100050496 | No / Not  publicly available | Unclear | Some concerns | Trial registration was reported, but no public protocol or  prespecified analysis plan was identified. |
