# Supplementary material for: Efficacy of transcranial magnetic stimulation for mild cognitive impairment: a systematic review and meta-analysis of randomized controlled trials
Source: Front Neurol. 2026 May 18;17:1788223. doi: 10.3389/fneur.2026.1788223 (PMC13222799; doi:10.3389/fneur.2026.1788223)
Supplement: Supplementary file 5 [file Table_5.docx]

**Supplementary Table 5.** Studies contributing to each outcome.

| **Domain** | **Outcome (instrument)** | **Metric** | **k** | **Total n** | **Contributing studies (first author, year)** | **Figure** |
| --- | --- | --- | --- | --- | --- | --- |
| Global cognition | MMSE | MD | 5 | 120 | Padala 2017; Song 2025; Wang 2023; and Zheng 2024 (three-arm trial counted as two comparisons) | Figure 3A |
|  | MoCA | MD | 6 | 337 | Liu 2025; Song 2025; Wang 2023; Wang 2025; and Zheng 2024 (three-arm trial counted as two comparisons) | Figure 3B |
| Executive/attention | Digit Span | MD | 6 | 297 | Cirillo 2023; Esposito 2022; Wang 2025; Fu 2025; and Zheng 2024 (three-arm trial counted as two comparisons) | Figure 4A |
|  | TMT-A (time) | MD | 5 | 108 | Padala 2017; Wang 2025; Song 2025; and Zheng 2024 (three-arm trial counted as two comparisons) | Figure 4B |
|  | TMT-B (time) | MD | 7 | 245 | Drumond 2015; Padala 2017; Wang 2025; Liu 2025; Song 2025; and Zheng 2024 (three-arm trial counted as two comparisons) | Figure 4C |
| Verbal Memory | AVLT immediate recall | MD | 3 | 111 | Cui 2019; Wang 2025; Song 2025 | Figure 5A |
|  | AVLT delayed recall | MD | 5 | 125 | Cui 2019; Wang 2025; Song 2025; and Zheng 2024 (three-arm trial counted as two comparisons) | Figure 5B |
|  | AVLT recognition | MD | 4 | 103 | Cui 2019; Wang 2025; and Zheng 2024 (three-arm trial counted as two comparisons) | Figure 5C |
| Language function | Semantic fluency | SMD | 6 | 134 | Drumond 2015; Cirillo 2023; Esposito 2022; Song 2025; and Zheng 2024 (three-arm trial counted as two comparisons) | Figure 6A |
|  | Naming ability | SMD | 3 | 69 | Cirillo 2023; Esposito 2022; Song 2025 | Figure 6B |
| Mood symptoms | Depression | SMD | 3 | 150 | Liu 2025; Cirillo 2023; Esposito 2022 | Supplementary Figure 2A |
|  | Anxiety | SMD | 3 | 150 | Liu 2025; Cirillo 2023; Esposito 2022 | Supplementary Figure 2B |

MMSE: Mini-Mental State Examination; MOCA: Montreal Cognitive Assessment; AVLT: Auditory Verbal Learning Test; TMT: Trail Making Test; MD: Mean differences; SMD: Standardized mean differences.
